# Bellevue College Medical Hospital

**Published:** 1877-04-20

**Authors:** 


BELLEVUE HOSPITAL MEDICAL COLLEGE.—See advertisement of this excellent Institution.

